# [^18^F]FDG PET/CT volumetric biomarkers for non-invasive prediction of HER2 expression in breast cancer patients

**DOI:** 10.22038/aojnmb.2024.79970.1563

**Published:** 2025

**Authors:** Mai Amr Elahmadawy, Heba Abdelhamed, Dina Hosny Gamal El-din, Mahitab Eltohamy, Adel Mohamed Ismail Hassan, Salwa Abd El-Gaid

**Affiliations:** 1 Nuclear Medicine Unit, National Cancer Institute (NCI), Cairo University, Cairo, Egypt; 2 Clinical oncology and nuclear medicine department, Zagazig University, Zagazig Egypt; 3 Radiology department , Kasr Al Aini medical school, Cairo University, Cairo, Egypt; 4 Pathology department, National Cancer Institute (NCI), Cairo University, Cairo, Egypt; 5 Surgical Oncology department, Ismailia teaching oncology hospital, Ismailia, Egypt

**Keywords:** PET/CT, HER2, Breast cancer

## Abstract

**Objective(s):**

to investigate the capability of ^18^F-fluorodeoxyglucose positron emission tomography/computed tomography ([^18^F]-FDG PET/CT) derived volumetric parameters to predict human epidermal growth factor receptor 2 (HER2) status in breast cancer patients.

**Methods:**

retrospective study enrolled 47 female patients with breast cancer. All patients had pretreatment [^18^F]-FDG PET/CT. Clinical data, pathology report and HER2 status were retrieved from medical records. In an attempt to assess the predictive value of the PET-derived metabolic parameters, Receiver operating characteristic (ROC) curve was constructed with area under curve analysis performed to detect best cutoff value of significant parameters for detection of HER2 positive.

**Results:**

No statistically significant difference was noted among both groups (HER2 positive and negative) in respect to age, menopausal status, histology, grade, T-stage, N-stage, or antigen Kiel 67 (Ki-67) index. ROC curve successfully marked cutoff point ≥42.35 for total lesion glycolysis (TLG) and  12.75 for metabolic tumor value (MTV) that are capable to discriminate positive versus negative HER2 expression in breast cancer patients with area under curve (AUC) 0.728 and 0.723 and P-values 0.002 and 0.004 respectively. Such cutoff point was not deduced for standard uptake value (SUV) max. Primary tumor TLG cutoff correlated well with age where 77.8% of patients with TLG  42.35 were older than 45 years old compared to 22.2% of them who were younger than 45 years, P-value0.047. Also 70.3% of patients with TLG exceeds  42.35 had T3 and 4 primary tumors while 65% of those with TLG <42.35 their primary tumors were T1 and 2, P-value0.03. As regards Primary tumor MTV cutoff point, significant correlations were noted in respect to T-stage where 78.2% of the patients with primary tumor MTV  12.75 were T3 and 4, compared to 66.6% of those with primary tumor MTV <12.75 were T1 and 2, P-value0.011.

**Conclusion:**

PET-derived volumetrics may serve as non-invasive predictors of biological processes represented here as HER2 expression in breast cancer patients.

## Introduction

 Breast cancer (BC) ranks the second most frequently diagnosed cancer in 2022, with an estimated 2.5 million new cases (11.6% of all cancer globally) and ranks fourth as a cause of cancer death at 6.9%. (1). Breast cancer has a heterogeneous nature with regard to molecular subtype. The human epidermal growth factor receptor 2 (HER2) status of BC contributes significantly to the heterogeneity of its different molecular subtypes (2).

 HER2 is a receptor tyrosine kinase that is positively expressed in approximately 10-15% of breast cancer cases (3). The clinical value of HER2 expression has been linked to the worse outcomes observed with positive group compared with HER2-negative BC patients. At the same time, these patients often benefit from HER2-targeted therapy such as trastuzumab, which significantly improves outcome (4).

 Therefore, it becomes crucial in clinical practice to determine pre-treatment HER2 status. HER2 status was mainly evaluated based on immunohistochemistry (IHC) and/or in situ hybridization (ISH) methods (5). These techniques rely on the accessibility of the biopsy. However, this approach can be limited, with the potential for false-negative results as the biopsy can represent only a small portion of the potentially heterogeneous lesion in multifocal tumors or even in the same tumor from so-called intratumor heterogeneity (6). It is the coexistence of multiple subsets of cancer cells that differ genetically, phenotypically, or behaviorally within the primary tumor or between the primary tumor and its metastases (6). Furthermore, tumor biology may change over time and in response to therapy, primarily due to epithelial-mesenchymal transition (7). 

 Thus, an effective and non-invasive method to predict HER2 expression and support further clinical management decisions is needed.

^18^F-fluorodeoxyglucose positron emission tomography/ [^18^F]-FDG PET/CT is a non-invasive imaging method widely used in oncology (8). [^18^F]-FDG PET/CT is able to accurately reflect aggressive tumor biology through putative enhanced glycolysis and glucose hypermetabolism in the tumor (8). It can also indicate the sample most representative of tumor aggressiveness for biopsy (9). 

 Previous studies have endorsed the predictive capabilities of metabolic [^18^F]-FDG PET/CT metrics in various solid and hematologic malignancies (10-12). Thus, quantitative parameters derived from PET images in terms of maximum standard uptake value (SUV_max_), total lesion glycolysis (TLG), and metabolic tumor value (MTV) can be valuable biomarkers to express the biological heterogeneity of breast cancer molecular subtypes. The aim of the current study is to investigate the capability of PET-derived metabolic and volumetric parameters to predict HER2 status in breast cancer patients.

## Methods

 This retrospective study enrolled 47 female patients with breast cancer between January 2013 and February 2018. All patients had pretreatment [^18^F]-FDG PET/CT scans. Clinical data, pathology report, hormone receptor status, HER2 status, Ki67, nodal status, and treatment strategies were retrieved from medical records. TNM staging was based on the American Joint Committee on Cancer (AJCC) staging srogram, 8th Edition (13).

 Patients had the following inclusion criteria: (i) Adult female patients with histo-pathologically proven breast cancer; (ii) Patients had initial [^18^F]-FDG PET/CT scan and did not receive chemotherapy or radio-therapy treatment before imaging.

 The study was approved by the institutional review board IRB number (11398-15-1-2024). Informed consent was waived by the ethics Committee. 

***[*** ^18^ ***F]-FDG PET/CT technique***

 PET/CT scanning was performed using Discovery PET-CT scanner (GE Healthcare, Milwaukee, Wisconsin, USA). Patients were asked to fast for 6 hours before the injection of [^18^F]-FDG. Activity of 370 MBq was administered. Blood glucose levels did not exceed 200 mg/ dL. Scanning started 60 min after tracer injection from the skull vault to mid-thigh with 6–8 bed positions (2 minutes / bed position). The CT was acquired using the following parameters: 120 kV, 140 mA, PITCH: 1.375, slice thickness: 3.75 mm. The images were reconstructed by iterative reconstruction with CT-based attenuation correction.

***PET/CT analysis***

 Two experienced nuclear medicine physicians with 18 and 15 years of experience interpreted the images, respectively. PET, CT and fused PET/CT images were reviewed at the dedicated workstation and software (E.soft; GE Medical Solutions), and automatically determine the contour of the PET-based lesion with cutoff values of 41%. Images interpretation were performed visually and semi-quantitatively. For semi-quantitative analysis, a spherical volume of interest (VOI) was drawn over the [^18^F]-FDG avid lesions. PET metrics were calculated in all PET scans taking a relative threshold of 41% of the the maximum SUV (SUV_max_), and SUV_max_, total lesion glycolysis (TLG), as well as metabolic tumor volume (MTV) in the VOI were recorded. SUV_max_ was defined as the maximum uptake in the VOI that reflects the maximum tissue concentration of FDG in the tumor, MTV was the volume of the VOI after the tumor segmentation and TLG was measured as the product of SUV_mean_ by the MTV.

***HER2 analyses***

**Figure 1:**
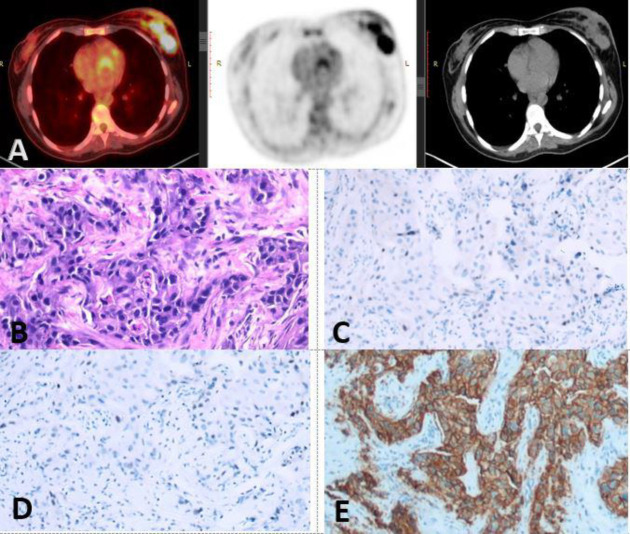
[^18^F]-FDG PET/CT fused, CT and PET images revealed: metabolically active left breast ill-defined soft tissue lesion measuring 4.1×2.5 cm with SUV_max_ 8.5, MTV 28.89 cm^3^ and TLG 152.55 SUV-bw×cm^3^ (**A**). Hx & E slide of a case of invasive duct carcinoma grade III (**B**), ER: Negative (Allred 0/8) (**C**), PR Negative, Allred (0/8) (**D**), HER-2: Positive score 3+, ×200 (**E**)

**Figure 2:**
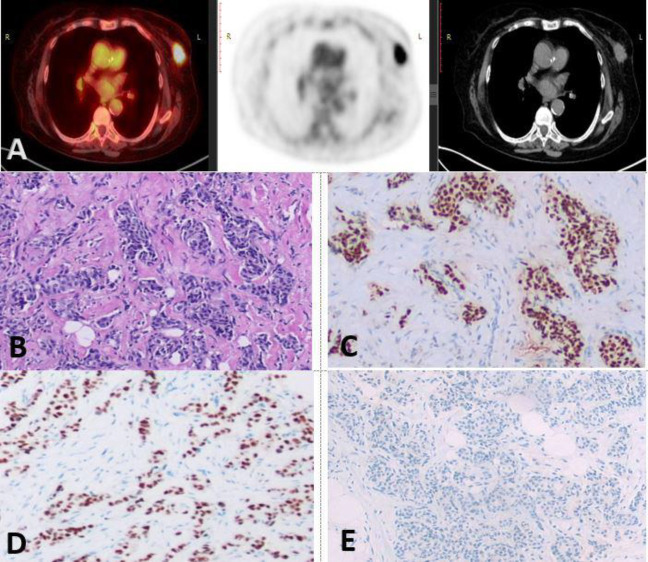
[^18^F]-FDG PET/CT fused, CT and PET images revealed: metabolically active left breast UOQ soft tissue lesion measuring 3.5×2.5 cm with SUV _max_ 6.43, MTV 7.01 cm^3^ and TLG 29.05 SUV-bw×cm^3^ (**A**). Hx&E stained slide of a case of invasive duct carcinoma, grade II, (**B**), ER: Positive, (Allred 8/8) (**C**) PR: Positive (Allred 8/8) (**D**), HER-2: Negative, score 0, ×200(**E**)

**Figure 3:**
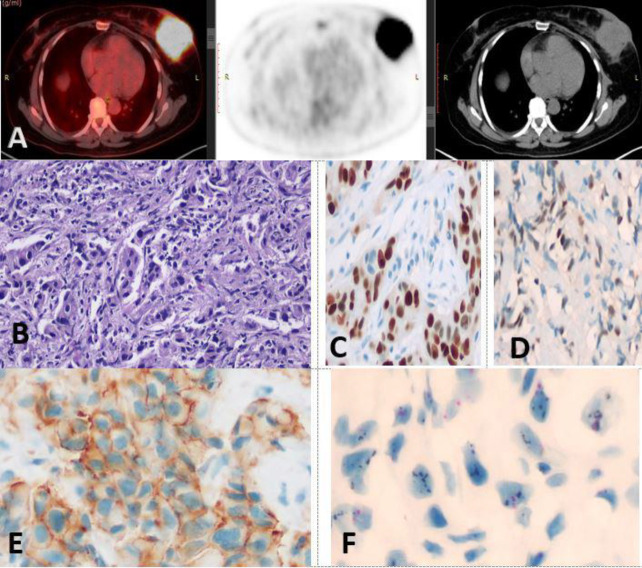
[^18^F]-FDG PET/CT fused, CT and PET images revealed: metabolically active left breast UOQ soft tissue lesion measuring 6.5×6 cm with SUV_max_ 23.9, MTV 68.04 cm^3^ and TLG 989.29 SUV-bw×cm^3^ (**A**). Hx&E-stained slide of a case of invasive duct carcinoma, grade III ×200(**B**), ER: Positive (Allred 8/8) (**C**), PR: Positive (Allred 5/8) (**D**), HER-2: Equivocal, score 2+ ×400(**E**), HER-2: Amplified with clusters by SISH, ×600(**F**)

 The evaluation of molecular subtypes of cases was carried out using immunohistochemical studies on tissue samples. HER2+ breast cancer is defined, according to the American Society of Clinical Oncology/College of American Pathologists (ASCO/CAP) guidelines (14), when a complete and intense circumferential membrane staining for the HER2 protein in >10 % of tumor cells (3+ score) is found at IHC and/or the HER2 gene is amplified at in situ immunofluorescence (ISH) techniques, with an HER2/CEP17 ratio ≥2.0 and an average HER2 gene copy number≥4.0 signals/cell (Figure 1, 2 and 3).

 Antigen Kiel 67 (Ki-67) expression was recorded as the percentage (ranging from 0% to 100%) of tumor cells showing positive nuclear staining. High Ki-67 expression was defined using a cutoff value of 30% according to the latest St Gallen meeting in 2021 (15).

***Statistical analyses***

 Data were coded and entered using the statistical package for the Social Sciences (SPSS) version 26 (IBM Corp., Armonk, NY, USA). Data was summarized using mean, standard deviation, median, minimum and maximum in quantitative data and using frequency (count) and relative frequency (percentage) for categorical data. Comparisons between quantitative variables were done using the non-parametric Mann-Whitney test (16). For comparing categorical data, Chi square ( 2) test was performed. Exact test was used instead when the expected frequency is less than 5 (17). 

 ROC curve was constructed with area under curve analysis performed to detect best cutoff value of significant parameters for detection of HER-2 +ve. P-values less than 0.05 were considered as statistically significant.

## Results

 Forty-seven female patients with breast cancer were enrolled in the current study. 43 patients (91.5%) of them had invasive duct carcinoma (IDC) and the remaining 4 (8.5%) patients had invasive lobular carcinoma (ILC). 

 31 (66.0%) of the group ≥45 years old. 23 (48.9%) were postmenopausal. 5 (10.6%) grade I, 21 (44.7%) grade II, and 21 (44.7%) grade III. 2 patients (4.3%) T1, 19 (40.4%) T2, 17 (36.2%) T3, 9 (19.1%) T4. Further HER2 analyses revealed 16 out of the 47 patients were HER2 positive representing 34% of the enrolled group. Different clinicopathological parameters were assessed. No statistically significant difference was noted among both groups (HER2 positive and negative) in respect to age, menopausal status, histology, grade, T-stage, N-stage, or Ki-67 index. For treatment 15 patients with HER 2 positive expression received anti-HER2 and 1 patient received hormonal treatment and those with negative expression 27 patients of them received chemotherapy, 1 received chemo- and hormonal therapies and 3 received only hormonal treatment (Table 1).

**Table 1:** Clinicopathological parameters in breast cancer patients with positive versus negative HER2 expression

**Clinico-pathological parameters**		**HER2 expression**				**P-value**
		**Positive**		**Negative**		
		**number**	**percentage**	**number**	**percentage**	
**Age**	**<45**	6	37.5%	10	32.3%	0.719
	**≥45**	10	62.5%	21	67.7%	
**Menopausal status**	**premenopausal**	8	50.0%	16	51.6%	0.917
	**postmenopausal**	8	50.0%	15	48.4%	
**Histology**	IDC*	16	100.0%	27	87.1%	0.284
	ILC*	0	0.0%	4	12.9%	
**Grade**	**I**	1	6.3%	4	12.9%	0.253
	**II**	10	62.5%	11	35.5%	
	**III**	5	31.3%	16	51.6%	
T- stage*	**T1**	1	6.3%	1	3.2%	0.962
	**T2**	7	43.8%	12	38.7%	
	**T3**	5	31.3%	12	38.7%	
	**T4**	3	18.8%	6	19.4%	
N-stage*	**N0**	2	12.5%	7	22.6%	0.503
	**N1**	10	62.5%	12	38.7%	
	**N2**	1	6.3%	2	6.5%	
	**N3**	3	18.8%	10	32.3%	
**Ki-67-labeling index**	**Low**	2	12.5%	3	10.3%	1
	**High**	14	87.5%	26	89.7%	
**Treatment**	**Chemotherapy**	0	0.0%	27	87.1%	< 0.001
	**Chemotherapy & Anti-HER2**	15	93.8%	0	0.0%	
	**Chemotherapy & Hormonal**	0	0.0%	1	3.2%	
	**Hormonal**	1	6.3%	3	9.7%	

IDC: invasive duct carcinoma

* ILC: invasive lobular carcinoma

* T- stage: tumor stage

* N-stage: nodal stage

 Pretreatment PET-derived metabolic parameters were significantly different among HER2 positive and negative groups. Higher TLG and MTV values were obtained in HER2 positive group with median values 87.6 and 20.1 respectively compared to 29.0 and 5.95 for HER2 negative group, p-values 0.011 and 0.013 respectively. Higher SUV_max_ was also noted among HER2 positive group yet did not reach statistical significance (Table 2).

**Table 2:** Pretreatment [^18^F]-FDG PET/CT derived metabolic parameters in breast cancer patients with positive versus negative HER2 expression

**Primary tumor metabolic parameters**	**HER2**										**P -value**
	**Positive**					**Negative**					
	**Mean**	**SD**	**Median**	**Min**	**Max**	**Mean**	**SD**	**Median**	**Min**	**Max**	
**SUV**_max_ *	12.92	9.2	9.7	3.3	38.5	9.04	3.34	9.3	3.6	17.3	0.508
TLG*	451.66	1382.3	87.6	2.0	7775.7	60.31	82.35	29.0	1.6	316.5	0.011
MTV*	46.38	121.99	20.1	0.7	692.3	11.18	15.03	5.95	0.7	57.9	0.013

* SUV: standard uptake value

* TLG: total lesion glycolysis

* MTV: metabolic tumor volume

**Figure 4:**
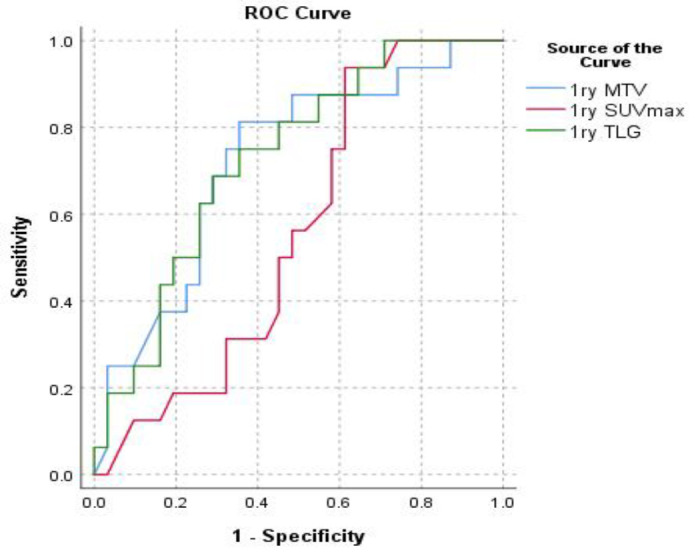
ROC for pretreatment [^18^F]-FDG PET/CT derived metabolic parameters cutoffs that discriminate positive versus negative HER2 expression in breast cancer patients

 In an attempt to assess the predictive value of the PET-derived metabolic parameters, ROC was used to mark cutoff points that are capable to discriminate positive versus negative HER2 expression in breast cancer patients. Cutoff point 42.35 for TLG and 12.75 for MTV were successfully marked with AUC 0.728 and 0.723 and p-values 0.002 and 0.004 respectively. Such cutoff point was not deduced for SUV_max_ (Table 3, Figure 4).

**Table 3:** ROC for pretreatment [^18^F]-FDG PET/CT derived metabolic parameters cutoffs that discriminate positive versus negative HER2 expression in breast cancer patients

**Primary tumor metabolic parameters**	**Area under curve**	**P value**	**95% Confidence Interval**		**Cut off value**	**Sensitivity %**	**Specificity %**
			**Lower Bound**	**Upper Bound**			
**SUV**_max_	0.559	0.477	0.395	0.723	----	----	----
**TLG**	0.728	0.002	0.582	0.874	42.35	68.8	71
**MTV**	0.723	0.004	0.569	0.876	12.75	81.3	64.5

*SUV: standard uptake value

* TLG: total lesion glycolysis

* MTV: metabolic tumor volume

 Deduced predictive cutoff points were furtherly tested in respect to different clinicopathological features. Primary tumor TLG cutoff correlated well with age where 77.8% of patients with TLG 42.35 were older than 45 years old compared to 22.2% of them who were younger than 45 years, P-value 0.047. Also 70.3% of patients with TLG exceeds 42.35 had T3 and T4 primary tumors while 65% of those with TLG <42.35 their primary tumors were T1 and T2, P-value 0.03. Such significant correlations were not observed with Histology, grade, N-stage, or Ki-67-labeling index (Table 4).

**Table 4:** pretreatment [^18^F]-FDG PET/CT derived TLG in respect to different clinicopathological parameters in breast cancer patients

**Clinicopathological parameters**		**Primary tumor TLG**				**P-value**
		**<42.35**		**42.35**		
		**Number**	**Percentage**	**Number**	**Percentage**	
**Age**	**<45**	10	50.0%	6	22.2%	0.047
	**≥45**	10	50.0%	21	77.8%	
**Histology**	IDC*	18	90.0%	25	92.6%	1
	ILC*	2	10.0%	2	7.4%	
**Grade**	**I**	2	10.0%	3	11.1%	0.164
	**II**	12	60.0%	9	33.3%	
	**III**	6	30.0%	15	55.6%	
T- stage*	**T1**	2	10.0%	0	0.0%	0.03
	**T2**	11	55.0%	8	29.6%	
	**T3**	6	30.0%	11	40.7%	
	**T4**	1	5.0%	8	29.6%	
N-stage*	**N0**	4	20.0%	5	18.5%	0.704
	**N1**	11	55.0%	11	40.7%	
	**N2**	1	5.0%	2	7.4%	
	**N3**	4	20.0%	9	33.3%	
**Ki-67-labeling index**	**Low**	2	10.0%	3	12.0%	1
	**High**	18	90.0%	22	88.0%	

* IDC: invasive duct carcinoma

* ILC: invasive lobular carcinoma

* T- stage: tumor stage

* N-stage: nodal stage

 As regards Primary tumor MTV cutoff point, significant correlations were noted in respect to T-stage where 78.2% of the patients with primary tumor MTV 12.75 were T3 and 4, compared to 66.6% of those with primary tumor MTV <12.75 were T1 and 2, P-value 0.011. No statistically significant correlations were observed in respect to age, histology, grade, N-stage or Ki-67-labeling index, p-values >0.05 (Table 5).

**Table 5:** pretreatment [^18^F]-FDG PET/CT derived MTV in respect to different clinicopathological parameters in breast cancer patients

**Clinicopathological parameters**		**Primary tumor MTV**				**P value**
		**<12.75**		**12.75**		
		**Number**	**Percentage**	**Number**	**Percentage**	
**Age**	**<45**	10	41.7%	6	26.1%	0.260
	**≥45**	14	58.3%	17	73.9%	
**Histology**	IDC*	22	91.7%	21	91.3%	1
	ILC*	2	8.3%	2	8.7%	
**Grade**	**I**	1	4.2%	4	17.4%	0.420
	**II**	12	50.0%	9	39.1%	
	**III**	11	45.8%	10	43.5%	
T- stage*	**T1**	2	8.3%	0	0.0%	0.011
	**T2**	14	58.3%	5	21.7%	
	**T3**	6	25.0%	11	47.8%	
	**T4**	2	8.3%	7	30.4%	
N-stage*	**N0**	6	25.0%	3	13.0%	0.386
	**N1**	12	50.0%	10	43.5%	
	**N2**	2	8.3%	1	4.3%	
	**N3**	4	16.7%	9	39.1%	
**Ki-67-labeling index**	**Low**	2	8.7%	3	13.6%	0.665
	**High**	21	91.3%	19	86.4%	

*IDC: invasive duct carcinoma

* ILC: invasive lobular carcinoma

* T- stage: tumor stage

* N-stage: nodal stage

## Discussion

 HER2 overexpression or gene amplification is associated with an aggressive breast cancer phenotype (3). However, this alteration has paved the way in favour of HER2-targeted therapy, such as trastuzumab therapy and prediction of breast cancer sensitivity to combinations of therapeutic agents becomes possible. Hence, this biomarker has been placed at the forefront of therapeutic testing for breast cancer (5).

 Meanwhile, some challenges may face invasive biopsy including known tumor heterogeneity and the selected biopsy site may not represent the most aggressive part, along with treatment-related dynamic subcellular changes that eventually occur leading to altered tumor biology (6). Therefore, non-invasive ancillary prediction technology has become paramount for management tailoring.

 HER2 has been reported to be overexpressed or amplifed in 15–30% of breast cancer cases (3). Similarly, in our study, breast cancer patients with HER2 positive expression represented 34% of the enrolled group.

 Though, clinicopathological parameters have been implicated among the factors influencing the heterogeneity of breast cancer (2). However, in current work no statistically significant difference was noted among both groups (HER2 positive and negative) in respect to age or pathological parameters. Previous study carried out by Esmat E. et al, revealed a significant correlation between HER2 positive expression and old age women, tumor size >5cm and tumor with grade 3 (18). On the contrary, Yadav R. et al, found No statistically significant association in positive/negative expressions of Her2 and different age groups, tumor grade, tumor size or histological types (19). Jang Y. et al, compared clinicopathologic characteristics between HER2-positive and HER2-negative patients with pure mucinous breast carcinoma. No statistically significant correlation was noted in respect to age. Meanwhile, HER2-positive group tumor size was larger, with higher nuclear and histologic grades and showed a more frequent high Ki-67 labeling index (20). 

 The rationale for the use of [^18^F]-FDG PET/CT in initial and therapy response assessment is based on the increased rate of glycolysis in different tumors compared with normal tissue and that FDG accumulates at a rate proportional to the tumor glucose utilization (8). The derived quantitative biomarkers stand behind [^18^F]-FDG PET/CT reliability as a non-invasive diagnostic and prognostic tool (21). 

 The standardized uptake value (SUV) is the most commonly used PET-derived semi-quantitative parameter as it represents the magnitude of [^18^F]-FDG avidity and reflects tissue proliferation (22). SUV_max_ is a sensitive indicator of metabolic activity and provides better reproducibility between scans. However, it represents a single-pixel value and does not reflect the whole tumor turnover and heterogeneity (23). Hence other volumetric parameters namely the metabolic tumor volume (MTV) and total lesion glycolysis (TLG) were also investigated in several tumors (9). 

 These metrics require delineation or segmentation of the FDG-avid lesions using a 3D region isocontour-based VOIs. The methods used for tumor segmentation are either fixed or adaptive (23). The challenge is to select the optimal fixed threshold to delineate the tumor taking into consideration the influence of the lesions size, tumor biology, and physiological background activity (23). In current study 3D isocontour at 41% of the maximum pixel value was used as recommended by the EANM procedure guidelines for tumour imaging: version 2.0 (24).

 Studying the relationships between [^18^F]-FDG PET-derived metrics and clinicopathological parameters in cancer patients has always been essential to aid precise management tailoring and anticipate the outcome (9). However, conflicting results have been reported regarding breast cancer. Groheux et al. found that SUV_max_ and TLG varied among breast different phenotypes (Her-2-positive, ER-positive/ HER-2-negative and triple negative) but none of the PET metrics provided high accuracy in distinguishing between prognostic subtypes of breast cancers (25). A significant relationship between hormone receptor/HER2 status and TLG has been reported by Kaida et al. They observed that TLG was a promising biomarker to indicate clinicopathological features and tumor metabolism better than SUV_max_ or MTV (26). 

 Meanwhile, Aktas et al. reported that SUV_max_ was the most relevant parameter that reflected molecular subtypes and Ki-67 index, while TLG was associated with T-size, N-stage and distant metastases (9)). In present study, pretreatment PET-derived volumetric parameters were significantly different among HER2 positive and negative groups. Higher TLG (with median value 87.6) and MTV (with median value 20.1) were obtained in HER2 positive group compared to median values of 29.0 and 5.95 respectively, for HER2 negative group (p-values=0.011 and 0.013 respectively). Higher SUV_max_ was also noted among HER2 positive group however without achieving statistical significance. These results support the theory that dysregulated expression of HER2 gene leads to increased cellular proliferation (27).

 Moreover, in current study, ROC successfully identified predictive cut-points for TLG (42.35) and MTV (12.75) that were able to discriminate positive and negative HER2 expression in breast cancer patients (p-value=0.002 and 0.004, respectively). 

 However, a discriminating cut-off point for SUV_max_ could not be obtained. This may reflect the superiority of MTV and TLG compared to SUV_max_ in mirroring the entire tumor biological process in breast cancer. Also, although SUV_max_ is a sensitive index of metabolic activity and tissue proliferation, it may also be subject to bias due to multiple factors such as those related to technique, tumor histopathology, and tumor size (28).

 Significant correlation was observed with the present study between high TLG (exceeding the deduced cutoff 42.35) and females older than 45 years old, p-value 0.047. The biological aging process entailing enhanced subcellular changes coupled with increased susceptibility to mutagens could be factors integrated in initiating and promoting the tumorigenic process (29). This observation was not found with respect to MTV, probably because TLG represents [^18^F]-FDG avidity magnitude and metabolically active volume of tumor side by side.

 Current study revealed an association between high tumor stages (T3 and 4) and high TLG and MTV values, which exceeded the discriminatory cut-off points (p-value 0.03, and 0.011 respectively). Chen S et al study also revealed a link between high TLG30% and high clinical stage and T classification, as well as multicentricity hence indicating high tumor burden and aggressiveness (30).

 Ki-67 antigen is a cell protein related to proliferation. It influences cell synthesis, metabolism and prognosis (14). Previous studies reported positive correlations between Ki67 expression and degree of [^18^F]-FDG uptake (31, 32).On the contrary, this study did not show direct association between volumetric parameters and Ki-67. This may be due to the infrequent representation of low Ki67 expression within our group and the relatively small population enrolled. In the same vein, no significant correlation between volumetric parameters and tumor histology or grade was also observed.

 Some limitations are noticed in the current study. First, the retrospective nature of a single centric experience with a relatively small population enrolled. Second, the tumoral heterogeneity and the assumption that other genes are also involved in breast cancer development such as c-myc gene amplification, which was not evaluated in the present work.

## Conclusion

 PET-derived volumetrics may serve as non-invasive predictors of biological processes represented here as HER2 expression in breast cancer patients. Thus, they may corroborate biopsy findings, provide a second opportunity to re-evaluate suspected pathological false-negative results due to tumor heterogeneity, and may even provide a predictive impression for equivocal HER2 cases. Hence, incorporating image-derived risk factors into the initial patient assessment may aid in the precise management of breast cancer.

## Statements and Declarations

The authors declare that no funds, grants, or other support were received.

No relevant financial or non-financial interests to disclose.

No conflict of interest.
